# A New Skeleton Model and the Motion Rhythm Analysis for Human Shoulder Complex Oriented to Rehabilitation Robotics

**DOI:** 10.1155/2018/2719631

**Published:** 2018-06-03

**Authors:** Song Zhibin, Ma Tianyu, Nie Chao, Niu Yijun

**Affiliations:** Key Laboratory of Mechanism Theory and Equipment Design of Ministry of Education, Tianjin University, Tianjin 300072, China

## Abstract

Rehabilitation robotics has become a widely accepted method to deal with the training of people with motor dysfunction. In robotics medium training, shoulder repeated exercise training has been proven beneficial for improving motion ability of human limbs. An important and difficult paradigm for motor function rehabilitation training is the movement rhythm on the shoulder, which is not a single joint but complex and ingenious combination of bones, muscles, ligaments, and tendons. The most robots for rehabilitation were designed previously considering simplified biomechanical models only, which led to misalignment between robots and human shoulder. Current biomechanical models were merely developed for rehabilitation robotics design. This paper proposes a new hybrid spatial model based on joint geometry constraints to describe the movement of the shoulder skeletal system and establish the position analysis equation of the model by a homogeneous coordinate transformation matrix and vector method, which can be used to calculate the kinematics of human-robot integrated system. The shoulder rhythm, the most remarkable particularity in shoulder complex kinematics and important reference for shoulder training strategy using robotics, is described and analyzed via the proposed skeleton model by three independent variables in this paper. This method greatly simplifies the complexity of the shoulder movement description and provides an important reference for the training strategy making of upper limb rehabilitation via robotics.

## 1. Introduction

Rehabilitation robots have received increasing interest to provide rehabilitative therapy following neurological injuries such as stroke and spinal cord injury [1]. The shoulder is one of the most complex motor function areas of the human body, which has a direct impact on the recovery of upper limb motor function. When a shoulder has motor dysfunction caused by injury or disease, it often needs reasonable rehabilitation suitable for the specific conditions of the patient. For this situation, some studies have shown that a rehabilitation strategy based on task orientation that fuses high intensity repetitive exercise training will be very conducive to the improvement of athletic ability, which is one of the most effective rehabilitation methods [2–7], while the upper limb rehabilitation robot is a universally acknowledged effective way to carry out the rehabilitation strategy.

There are lots of robotics proposed currently for lower limb and upper limb rehabilitation. Most of them got fruitful achievement even though accurate motion information of bones and muscles was not considered thoroughly. There have been many attempts in robotics which evolved from a simplified 3-degrees of freedom (DoF) ball and socket, such as Carignan and Liszka [8] who have defined the shoulder as a 3-DoF ball-and-socket joint. However, it is not effective for a shoulder rehabilitation using exoskeleton robotics which has three orthogonal axes intersecting at one point to assume shoulder as a ball-and-socket joint, because the shoulder cannot be regarded as a single joint but complex and ingenious combination of bones, muscles, ligaments, and tendons. The remarkable characteristic of the shoulder complex is the movement rhythm involving the motion of the scapula, clavicle, and humerus. The rhythm analysis is important and necessary for shoulder complex rehabilitation training.

Biomedical researchers have done a lot of analysis on musculoskeletal models for shoulder training. In 1965, Dempster [9] established a simple two-dimensional series link model to describe the relative motion characteristics among bones of the shoulder; however, the model only has existing qualitative description, lacking the description with definition and quantitative analysis of the joint type. Engin and Chen [10] established a three-dimensional 6-DoF rigid-body model of the humerus relative to the human torso in 1986. Furthermore, Engin and Tumer increased the degrees of freedom of the shoulder complex model to 9 in 1989. Although Engin and Chen established a three-dimensional model of shoulder bones to analyze their kinematic characteristics, the series connection of the shoulder, without exception, simplified the scapula into two connecting rods that are connected to the clavicle and humerus.

There are complicated models in the shoulder joint. Garner and Pandy [11] established a 13-DoF human upper limb skeleton model in 1999. The model does not define the geometric constraints of the scapulothoracic articulation joint (ST) nor analyze the motion of the model. Based on the concept of this model, Maurel and Thalmann and Maurel et al. [12, 13] treat the ST joint as a single point contacting with the surface with 5 DoFs, but the study lacks systemic analysis. Tondu [14] proposed a similar shoulder mechanism model in 2005, which defines the ST joint as a planar subordinate on the basis of the Maurel model and ignores the rotational freedom of the sternoclavicular joint (SC) along the direction of the clavicle axis, modeling the shoulder girdle as a 2-DoF spatial parallel mechanism. This model simplifies the difficulty of analyzing shoulder girdles. In order to make the shoulder get a larger workspace and more activity, Lenarcic and Stanisic [15] in 2006 proposed a shoulder girdle skeleton system using a six-legged six-joint parallel mechanism to deal with the simulation, but it is difficult to determine the position of each joint and the length of the parallel bars corresponding to the actual shoulder girdle. It is also difficult to correspond to the movement of a single individual bone.

The current rehabilitation robots were designed with a simplified skeleton model and set the degrees of freedom such as the robotic-arm exoskeleton proposed by Klein et al. [16]. Therefore, this paper provides a suitable model that describes the shoulder complex motion via a model of a spatial hybrid mechanism with four rods and four joints and verifies the accuracy of the model through real data for rehabilitation robots in the shoulder joint. Among them, SC joint, acromioclavicular (AC) joint, and glenohumeral (GH) joint are all defined as spherical hinge joints, and the thorax is approximated as an ellipsoid. The constraint of the ST joint is defined as the two fixed points on the suprascapular connected to the thorax ellipsoid in a point contact constraint, and the rotation of the SC joint around the clavicular axis is considered to be an extra DoF.

## 2. Modeling and Analysis

In the model of the shoulder musculoskeletal dynamics, the posture analysis of the bones constituting the shoulder is the basis for shoulder motion analysis, muscle drive characteristics, muscle strength, and joint force analysis. Due to the complexity of the shoulder skeletal system, it is difficult to obtain the people's posture of the shoulder bone quickly by using the commonly used real-time skeleton marking method. Based on results provided by Garner and Pandy [11, 17] for the anatomical results of human shoulder geometry, the human shoulder skeleton system is simplified as a hybrid spatial mechanism model to simulate and analyze the movement of the shoulder bone. The motions of each joint and bone are described by defining the local coordinate systems fixed on the corresponding bones, and the position inverse analysis of the mechanism is completed by the homogeneous coordinate transfer matrix and vector method, which simplified the analysis and prediction of the shoulder skeleton configuration. Finally, this chapter verifies the rationality of the model through the experiment of the actual human shoulder.

### 2.1. A Proposed Model of Shoulder Skeleton System

The kinematics of the shoulder of the human body mainly depends on the skeleton system composed of the shoulder girdle and humerus compared with the muscle system and the tendon system, so in this paper, the skeleton system for the shoulder is mainly discussed. It can be considered a closed-loop rigid system composed of the thorax, clavicle, and scapula. Joints involved in shoulder movement involve the AC joint, the SC joint, the ST joint, and the GH joint.

**Figure 1:**
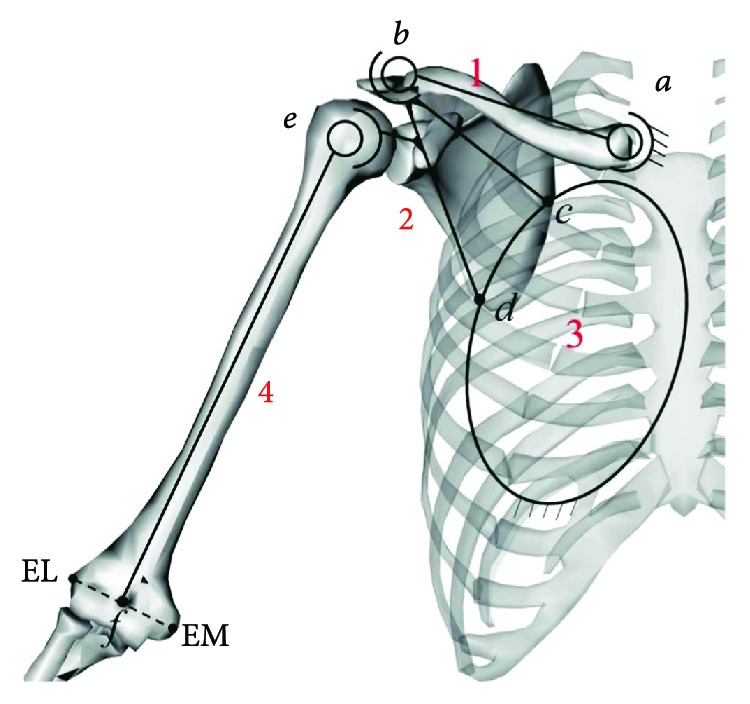
Schematic diagram of the shoulder mechanism.

In this paper, a hybrid spatial mechanism model is used to simulate the shoulder skeletal system, as shown in Figure 1. In this model, the SC joint, the AC joint, and the GH joint are denoted as *a*, *b*, and *e*, respectively, and are treated as the ideal 3-DoF ball-and-socket joint. The scapula achieves two-point contact with the thoracic ellipsoid surface through its medial lateral edge *c* and *d*, which may be equivalent to cylindrical-planar pairs with 4 DoFs. Among them, components 1, 2, 3, and 4 represent the clavicle, the scapula, the thoracic surface, and the humerus of the upper arm, respectively. The long axis of the humerus represents component 4, that is, the direction of connecting the center of the GH joint *e* and the center of the elbow *f*, where the elbow joint *f* is defined as the midpoint of the outer lateral ankle EL and the inside ankle EM.

### 2.2. Kinematics Analysis of the Proposed Model

In the above mechanism model, components 1, 2, 3, and 4 constitute a hybrid spatial mechanism containing a closed chain and an open chain, where components 1, 2, and 3 form a closed-loop chain.

#### 2.2.1. To Calculate DoFs of the Proposed Mechanism

To obtain the kinematics of the mechanism, we should first analyze its DoFs. For the closed loop of the shoulder girdle, the DoF of the mechanism (*M*) is obtained by the Kutzbach-Grubler formula [18].

(1) $$\begin{array}{c} M=6\left(n-g-1\right)+\overset{g}{\underset{i=1}{\sum }}f_{i}, \end{array}$$

where *n* is the number of mechanism components, *g* is the number of joints, and *f*_*i*_ is the relative freedom of the *ith* motion pair. The closed-loop part has three components and three motion pairs. Both SC and AC are ball-and-socket joints with 3 DoF. The ST joint is a cylinder-plane pair with 4 DoF. So a closed chain with 4 DoF in the shoulder joint system can be obtained. Considering the humerus movement, the total degree of freedom of the shoulder is up to 7. However, in the process of activity of the shoulder, the rotation of the clavicle around its axis is very small and it has an internal DoF that does not change the whole posture of link 4 [19–21]. Therefore, for joint *a*, the rotation in the direction of the winding of component 2 is an extra freedom. If the rotation angle is known, the motion posture of the shoulder girdle can be obtained by inputting three joint variables.

#### 2.2.2. To Establish Shoulder Global Coordinate and Local Coordinates Fixed to a Single Bone

**Figure 2:**
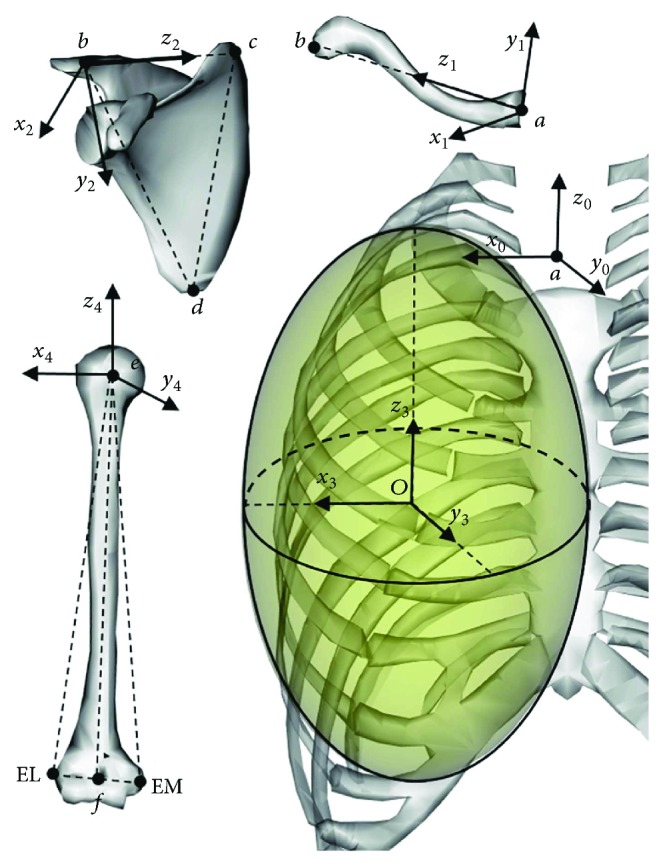
Shoulder bony segments and related reference frames.

In order to describe and analyze the kinematics of the skeleton model of the shoulder complex, every bone is fixed to one coordinate system, as shown in Figure 2. The origin point of the global coordinate system *S*_*o*_ = {*a* − *x*_0_*y*_0_*z*_0_} is the SC joint at point *a*, and axes *x*_0_, *y*_0_, and *z*_0_ are, respectively, parallel to three intersections of the human anatomical coronal plane, the sagittal plane, and the transverse plane. For the clavicle system *S*_1_ = {*a* − *x*_1_*y*_1_*z*_1_}, the origin is located on the SC joint at point *a*. The *z*_1_ axis is in the direction of the clavicle axis, and the *x*_1_ axis is defined on the horizontal plane and perpendicular to *z*_1_. For the scapular system *S*_2_ = {*b* − *x*_2_*y*_2_*z*_2_}, its origin point is located at the point of the AC joint at *b*, where the *z*_2_ axis is in the direction from *b* to the medial margin of the scapula *c* and the direction of *x*_2_ is perpendicular to the plane determined by points *b*, *c*, and *d*. *S*_3_ is parallel to *S*_0_. For the humeral coordinate system *S*_4_ = {*e* − *x*_4_*y*_4_*z*_4_}, the origin point is located at the center *e* of the glenoid joint, where the *z*_4_ axis is in the direction from *f* to *e*; the *y*_4_ axis is perpendicular to the plane determined by point *e*, EL, and EM; and the *x*_4_ axis is determined by the right-hand rule.

#### 2.2.3. Kinematics Analysis of the Joints in the Shoulder

In the shoulder girdle, the thorax, the clavicle, and the scapula are interconnected with each other through the joints SC, AC, and ST, thus forming a closed-loop mechanism. For the closed-chain mechanism, position analysis can be attributed to determine the remaining joint variables and skeletal posture using the known mechanism geometric parameters and at least three joint variables as inputs. In this paper, the purpose of inverse analysis is to verify the accuracy of the actual shoulder movement predicted by the model.

In order to describe the matrix conveniently, the distance between joint *a* and joint *b* is set as *l*_1_, the distance between the center of joint *b* and point *c* is *l*_2_, the distance between *b* and point *d* is *l*_3_, and the length of the humerus is *l*_4_.

According to the above definition for the coordinate system, transition from the system *S*_*o*_ = {*a* − *x*_0_*y*_0_*z*_0_} to *S*_1_ = {*a* − *x*_1_*y*_1_*z*_1_}, namely, the motion of the thorax relative to the thorax, can be expressed via the following matrix:

(2) T10=Rotz,θ1Roty,θ2Rotz,θ3=cθ1cθ3−sθ1cθ2sθ3−cθ1sθ3−sθ1cθ2cθ3sθ1sθ20sθ1cθ3+cθ1cθ2sθ3−sθ1sθ3+cθ1cθ2cθ3−cθ1sθ20sθ2sθ3sθ2cθ3cθ200001,

where s and c denote sine and cosine, respectively.

The conversion from system *S*_1_ to system *S*_2_ can be assumed to have moved *l*_1_ along the *z*-axis firstly and rotated *θ*_4_ around the *y*-axis, then rotated *θ*_5_ around the *x*-axis, and finally rotated *θ*_5_ around the *y*-axis. The conversion matrix is

(3) T21=Transz,l1Roty,θ4Rotx,θ5Roty,θ6=cθ4cθ6−sθ4cθ5sθ6sθ4sθ5cθ4sθ6+sθ4cθ5cθ60sθ5sθ6cθ5−sθ5cθ60−sθ4cθ6−cθ4cθ5sθ6cθ4sθ5−sθ4sθ6+cθ4cθ5cθ6l10001.

The position and scale of the thoracic ellipsoid are known. Assuming that the position of the ellipsoid center point *O* in the fixed system *S*_0_ is ^0^*P*_*O*_ = [*x*_0_, *y*_0_, *z*_0_, 1]^*T*^, the transfer matrix of ellipsoid *S*_3_ relative to fixed system *S*_0_ is

(4) T30=100x0010y0001z00001.

**Figure 3:**
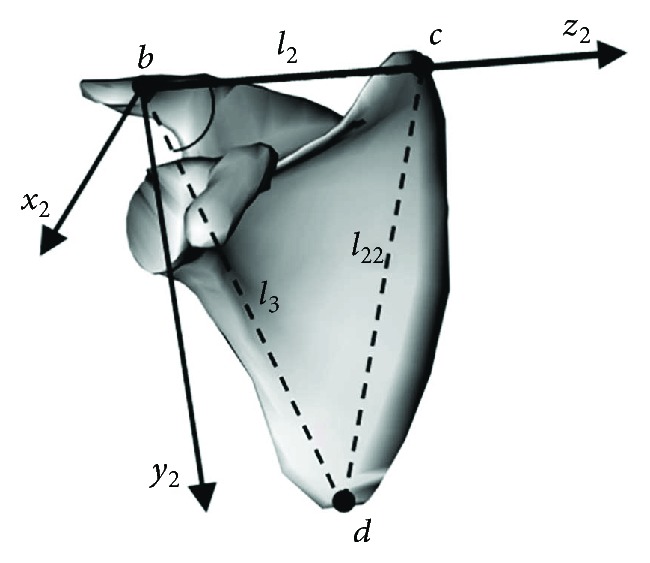
Points *c* and *d* located on the scapula.

If the semilong axes of the ellipsoid are, respectively, *m*, *n*, and *p* and point *c* and point *d* are located in the thoracic ellipsoid table surface, the position coordinates can be represented by the parametric equation of the ellipsoid:

(5) Pc3=msinφccosφcnsinφccosφcpcosφc1T,

(6) Pd3=msinφdcosφdnsinφdcosφdpcosφd1T,

where *φ*_*c*_, *ϕ*_*c*_, *φ*_*d*_, and *ϕ*_*d*_ in the formula, respectively, describes the position parameters of point *c* and point *d*. Point *c* and point *d* are also located on the shoulder blade, as shown in Figure 3. The position coordinates of *c* and *d* in the scapula system are

(7) Pc2=00l21T,Pd2=0l3sinγl3cosγ1T,

where *γ* in the formula is the included angle of $\vec{bc}$ and $\vec{bd}$, because points *b*, *c*, and *d* are all on the scapula, and *l*_2_, *l*_3_, and *γ* are constant. In summary, using the location transform relationship of *c* and *d*, the establishment of displacement constraint equation is as follows:

(8) Pc0=T10T221pc=T330pc,Pd0=T10T221pd=T330pd.

Formula (8) above can be expressed as follows:

(9) T10T221pc−T330pc=0,T10T221pd−T330pd=0.

Equation (9) is the joint position solution constraint equation of the girdle mechanism. Let each component be zero; the equations can be transformed into 6 constraint equations which contain a total of *θ*_1_ ~ *θ*_6_, *φ*_*c*_, *ϕ*_*c*_, *φ*_*d*_, *ϕ*_*d*_ 10 joint variables. Clavicle rotation around its own axis *z*_1_ is an extra degree of freedom, and if *θ*_3_ is zero, just any three rotational variables can be used as inputs to solve the remaining variables.

The kinematic analysis of the shoulder girdle is completed, while the humerus is connected to the shoulder complex through the GH joint as the end of execution. The motion posture of the upper arm is usually studied in the global reference system, using the ISB standard recommended by Wu and Cavanagh [22, 23] to describe the posture of the humerus. The rotation matrix applied to convert the global system *S*_0_ into the humerus system *S*_4_ is

(10) R40=Roty,θ7Rotx,θ8Rotz,θ9=cθ7cθ9−sθ7cθ8sθ9sθ7sθ8cθ7cθ9+sθ7cθ8sθ90sθ8sθ9cθ8−sθ8cθ90−sθ7cθ9−cθ7cθ8sθ6cθ7sθ8−sθ7cθ9+cθ7cθ8sθ900001.

If the position of the GH center coordinates Pe0=xeyeze1T is given, the conversion matrix for the translation from the humeral system *S*_0_ to the global system *S*_4_ is

(11) $$\begin{array}{c} \left[\begin{array}{cccc} c\theta_{7}c\theta_{9}-s\theta_{7}c\theta_{8}s\theta_{9} & s\theta_{7}s\theta_{8} & c\theta_{7}c\theta_{9}+s\theta_{7}c\theta_{8}s\theta_{9} & x_{e}\\ s\theta_{8}s\theta_{9} & c\theta_{8} & -s\theta_{8}c\theta_{9} & y_{e}\\ -s\theta_{7}c\theta_{9}-c\theta_{7}c\theta_{8}s\theta_{6} & c\theta_{7}s\theta_{8} & -s\theta_{7}c\theta_{9}+c\theta_{7}c\theta_{8}s\theta_{9} & z_{e}\\ 0 & 0 & 0 & 1 \end{array}\right]. \end{array}$$

### 2.3. Verification for Shoulder Skeletal Motion Based on Reference Data

In this paper, the size of shoulder bones refers to the anatomical data of human upper arm bones and joints obtained by Garner and Pandy [11, 17, 24]. Shoulder Database V1.1, published by Bolsterlee et al. [21] in 2014, is used as a reference source of shoulder motion data. Then, through the joint displacement constraint equations obtained above, the movement of the mechanism is analyzed by using Matlab.

#### 2.3.1. Shoulder Mechanism Geometry

In this paper, the size of the mechanism is based on the anatomy of human bones and joints obtained by Garner and Pandy; the size of each component shown in Figure 1 is as shown in Table 1 below. The semimajor axis of the thorax ellipsoid and the center point of its ellipsoid are shown in Table 2. The coordinates of the center point are in the fixed system *S*_0_.

The position of GH joint center in the scapular *S*_2_ is pe2=−0.2943.713.411T.

#### 2.3.2. Description of Shoulder Joint Movement

**Figure 4:**
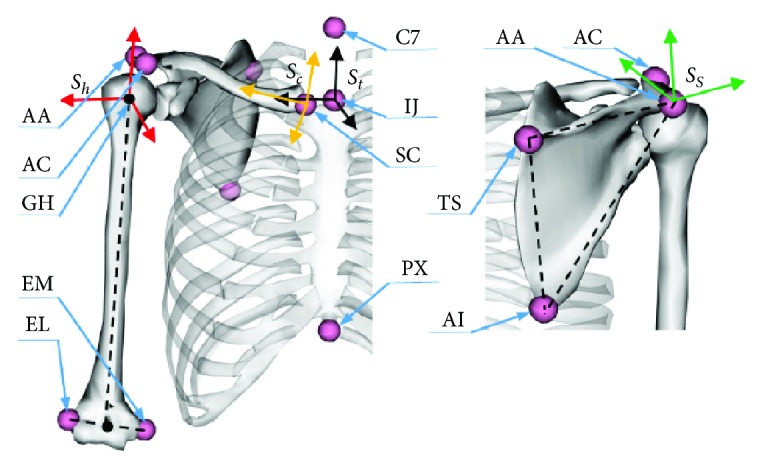
The bony landmarks and local coordinate systems defined in ISB.

The local coordinate system of each skeleton in Figure 2 is defined to be calculated conveniently. The ISB standards recommended by Wu and Cavanagh and Wu et al. [22, 23] are widely used in biomedical engineering to describe the human upper body bones and joints in the movement. In the ISB standard, the local coordinate system of the shoulder bones and the bony landmark are shown in Figure 4.

In this paper, the shoulder skeletal configuration analysis results and experimental measurements which are unified according to the ISB standard will be compared; therefore, it is necessary to figure out the corresponding relationship between the calculation of the coordinate system and the ISB coordinate system. Klein Breteler et al. [25] obtained a more favorable shoulder morphological datasets by dissecting the body of a 57-year-old male and establishing a universal dataset of human shoulder bones.  Table 3 shows the relevant parameters of bone markers and the size of the thorax of the general model and the experimental subjects in this article. All the coordinates are converted corresponding to the thorax system; the length unit is mm.

Through the definition of each local coordinate system and the characteristic sizes of each marked point of the shoulder skeleton in Tables 2 and 3, the relationship between the coordinate systems *S*_0_, *S*_1_, *S*_2_, and *S*_4_ used in the above calculation and each coordinate system *S*_*t*_, *S*_*c*_, *S*_*s*_, and *S*_*h*_ recommended in ISB standard can be described by the following rotation transformation matrix:

(12) R0t=010001100,

(13) R1c=100010001,

(14) R2s=0.95990.0373−0.2778−0.0131−0.9841−0.1773−0.2800−0.1738−0.9441,

(15) R4h=010001100.

#### 2.3.3. Definition of Shoulder Experimental Motion

**Figure 5:**
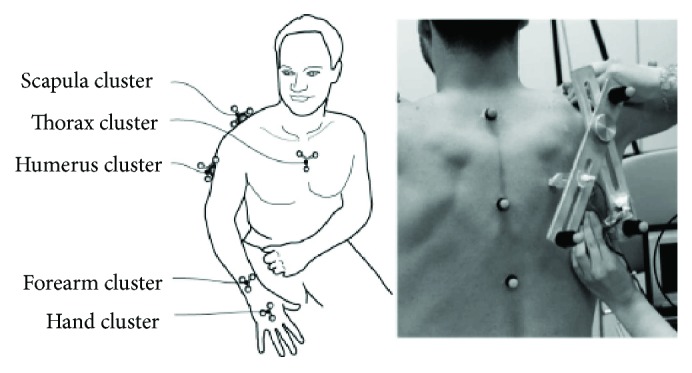
Placement of clusters of Optotrak markers.

Shoulder Database (Shoulder Database V1.1) published by Bolsterlee et al. in 2014 provides data of shoulder motion which has been used in the analysis [21, 26]. The experiment collected data from five healthy adult males. This article selects one of the objects *S*_1_ (age 29, height 186 cm, and weight 109 kg) from the database. The experiment used a set of motion detection system (Optotrak System, Northern Digital Inc., Waterloo, Ontario, Canada) to detect the spatial positions of six marker groups fixed to the thorax, scapula, humerus, forearm, and palm shown in Figure 5. The locations of the bone markers are arranged according to ISB standard, and the series of the position of the markers are collected at a frequency of 100 Hz.

In order to get accurate shoulder movement data, during the experiment, the subject was asked to complete the range of motion (ROM) tasks including shoulder abduction (ABD), flexion (FLEX), and scapular plane movement (SCAP) which contain all the basic movement of the shoulder shown in Table 4. With processing, the data of the movement posture of each bone in the shoulder can be uniformly converted into the value in *S*_*t*_.

#### 2.3.4. The Solution and Verification for Posture and Position of Each Bone

Through the processing of the spatial coordinates of each marker measured in the experiment, the joint variables *θ*_1_, *θ*_2_, *ϕ*_*d*_ are obtained, and the three independent joint displacements are used as input, and then the remaining six joint variables *θ*_4_, *θ*_5_, *θ*_6_, *φ*_*d*_, *ϕ*_*c*_, *φ*_*c*_ are obtained, as well as the shoulder girdle movement gesture, which is finally compared with the experimental measurement results.

**Figure 6:**
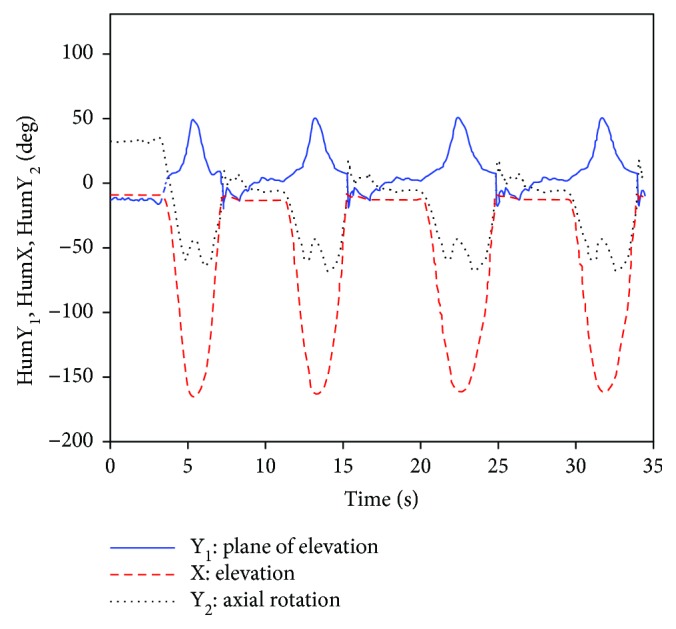
Movement posture of the humerus (ABD).

**Figure 7:**
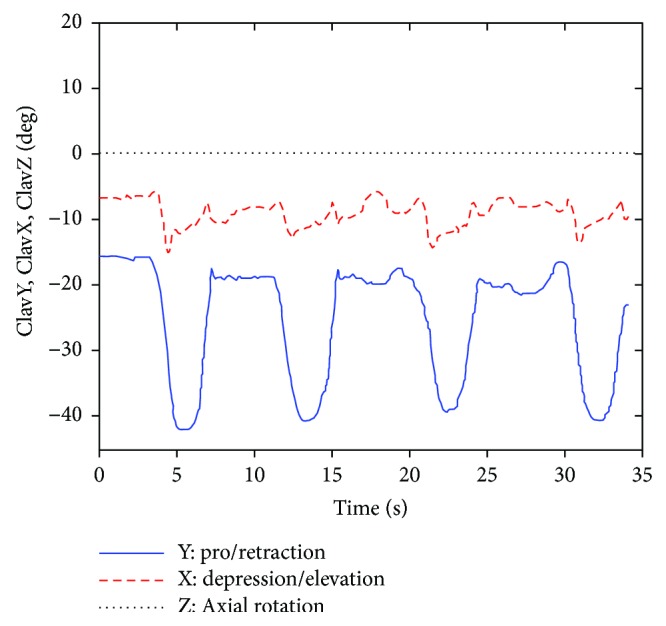
The posture of the clavicle that is adopted to be input parameters (ABD).

Figure 6 shows the thorax movement gesture of the experimental subject *S*_1_ in the ABD exercise test, and the posture of the humerus under YXY rotation sequence is described by three corresponding Euler angles HumY_1_, HumX, and HumY_2_.  Figure 7 shows the measured posture of the clavicle relative to the thorax, and according to ISB standard, the posture of the clavicle is described under YXZ rotation sequence. The three Euler angles formed that depended on them are ClavY, ClavX, and ClavZ, respectively.

Thus, at any time, the posture matrix of the clavicle relative to the thorax can be obtained:

(16) Rct=Roty,ClavYRotx,ClavXRotz,ClavZ.

Suppose that

(17) Rct=utcxvtcxwtcxutcyvtcywtcyutczvtczwtcz.

Substitute (17) into (12) and (13):

(18) R10=R0t−1RctR1c=uzvzwzuxvxwxuyvywy.

Let the third column of (18) and (2) be the same, and the following three constraint equations can be obtained:

(19) $$\begin{array}{c} s\theta_{1}s\theta_{2}=w_{z},\\ -c\theta_{1}s\theta_{2}=w_{x},\\ c\theta_{2}=w_{y}, \end{array}$$

then

(20) $$\begin{array}{c} \theta_{1}=\tan^{-1}\left(-\frac{w_{z}}{w_{x}}\right),\\ \theta_{2}=\tan^{-1}\left(\frac{w_{z}}{w_{y}s\theta_{1}}\right). \end{array}$$

**Figure 8:**
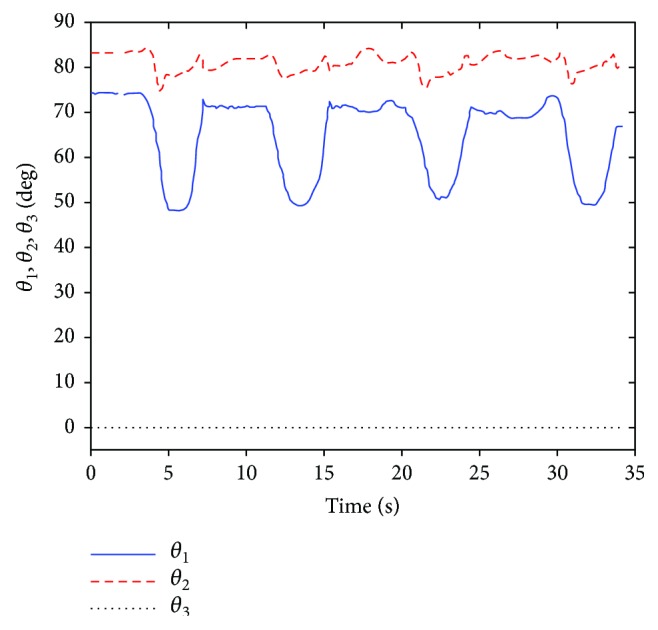
The joint angles *θ*_1_ and *θ*_2_ that are adopted to be input parameters (ABD).

Figure 8 is the result of the input joint variables *θ*_1_ and *θ*_2_.

Assuming that the object *S*_1_ is at a certain moment of ABD movement, the position vector of the sternoclavicular joint marking point SC is ^*t*^**p**_sc_, and the conversion relationship between *S*_*t*_ and *S*_0_ can be expressed as the following transfer matrix:

(21) T0t=R0t3×3000psct4×1.

Thus, the position vector of the lower scapular AI in the ellipsoidal system *S*_3_ can be obtained:

(22) pAI3=T3t−1pAIt=xAI,3yAI,3zAI,31T.

Combining formulas (4) and (12) and Table 3 on the thorax data, we can have the following formula:

(23) T3t=T0tT30=010−61.6001−148.110000001.

**Figure 9:**
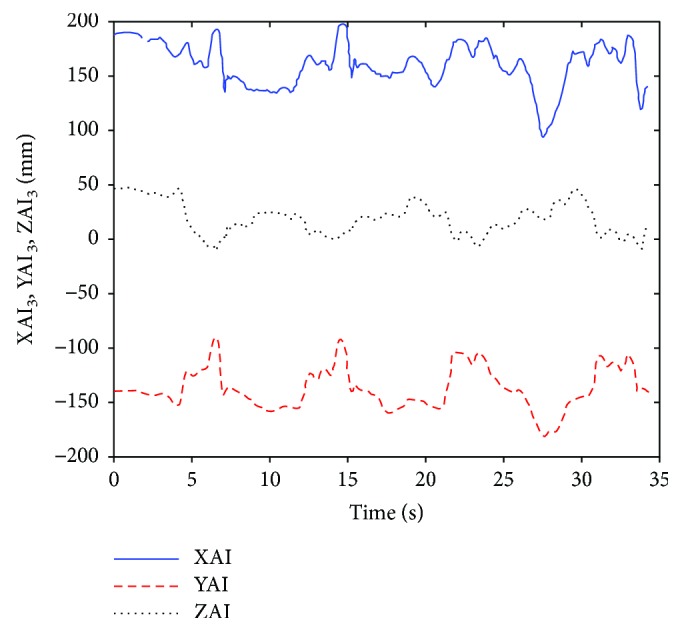
The position vector of AI described in ellipsoid frame *S*_3_.

In the ABD exercise of the experimental subject *S*_1_, the position of the lower scapular angle AI in the ellipsoid *S*_3_ is shown in Figure 9.

**Figure 10:**
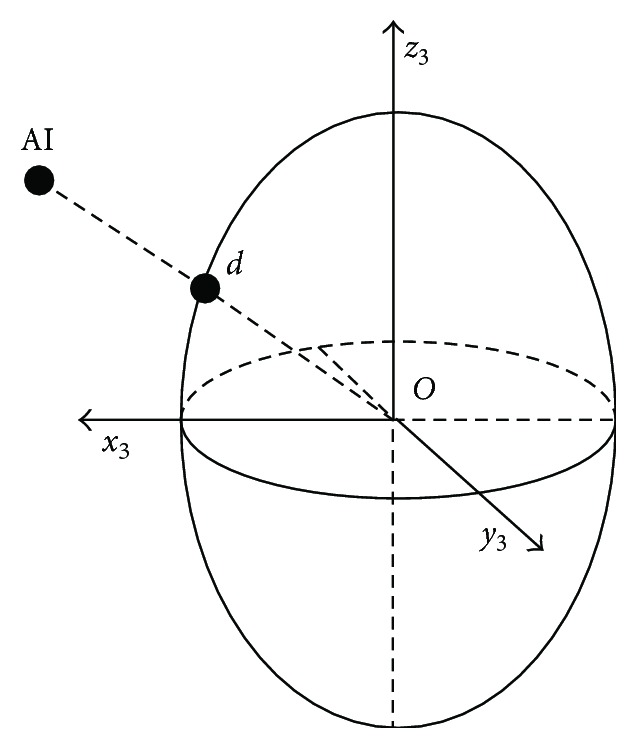
Point AI projects on the elliptical surface.

However, the fact is that the AI point is located on the shoulder blade but not always on the surface of the ellipsoid, which means it does not exactly coincide with point *d* in the model. In this paper, the projection point of the subscapular AI along the centerline of the ellipsoid is regarded as the equivalent point *d*. As shown in Figure 10, the connection between AI and ellipsoid center *O* intersects at *d* on the ellipsoid surface, and the position and size of the chest ellipsoid of the experimental subject *S*_1_ refer to Table 3.

It can be obtained that $\vec{Od}\left\|\vec{OAI}\right.$. Substitute (6) and (22), then there is

(24) $$\begin{array}{c} \frac{b\sin\varphi_{d}\sin\phi_{d}}{a\sin\varphi_{d}\cos\phi_{d}}=\frac{y_{AI,3}}{x_{AI,3}}. \end{array}$$

Another input joint variable can be found:

(25) $$\begin{array}{c} \phi_{d}=\tan^{-1}\left(\frac{a}{b}\frac{y_{AI,3}}{x_{AI,3}}\right). \end{array}$$

**Figure 11:**
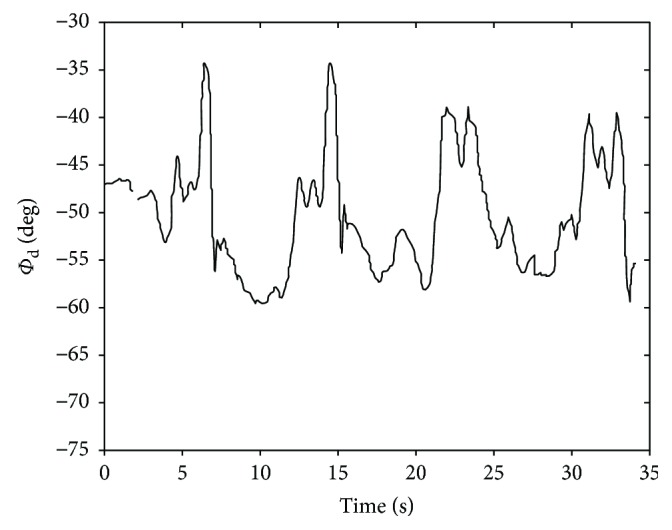
The joint angle *ϕ*_*d*_ that adopted to be the input parameter (ABD).

For the summary, for the object *S*_1_ in the ABD motion experiment, the input joint variable *ϕ*_*d*_ for the position analysis of the mechanism is shown in Figure 11.

**Figure 12:**
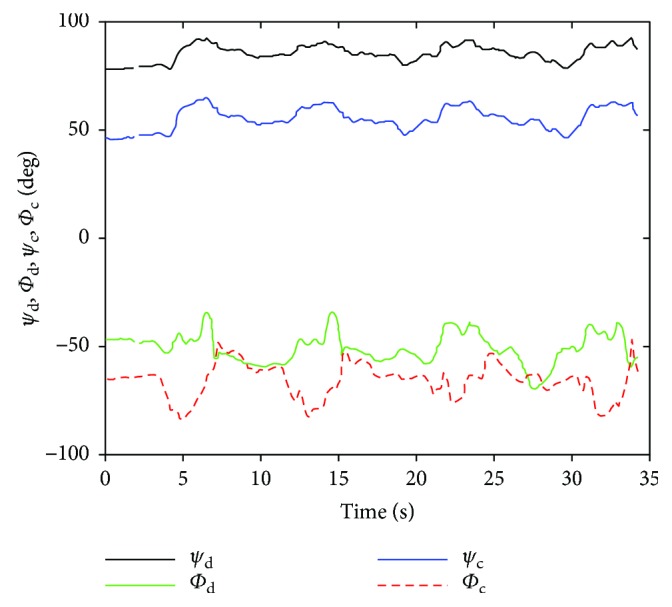
The joint angle *φ*_*d*_, *φ*_*c*_, *ϕ*_*c*_ (ABD).

**Figure 13:**
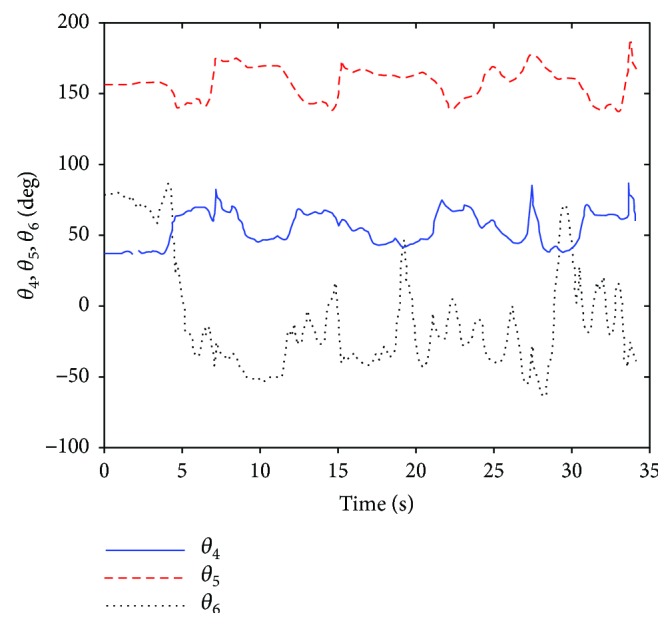
The curve of joint angle *θ*_4_, *θ*_5_, *θ*_6_ (ABD).

Then, entering the joint variables *θ*_1_, *θ*_2_, *ϕ*_*d*_ into closed solution equations, the joint variables *φ*_*d*_, *φ*_*c*_, *ϕ*_*c*_ and *θ*_4_, *θ*_5_, *θ*_6_ are obtained as shown in Figures 12 and 13.

**Figure 14:**
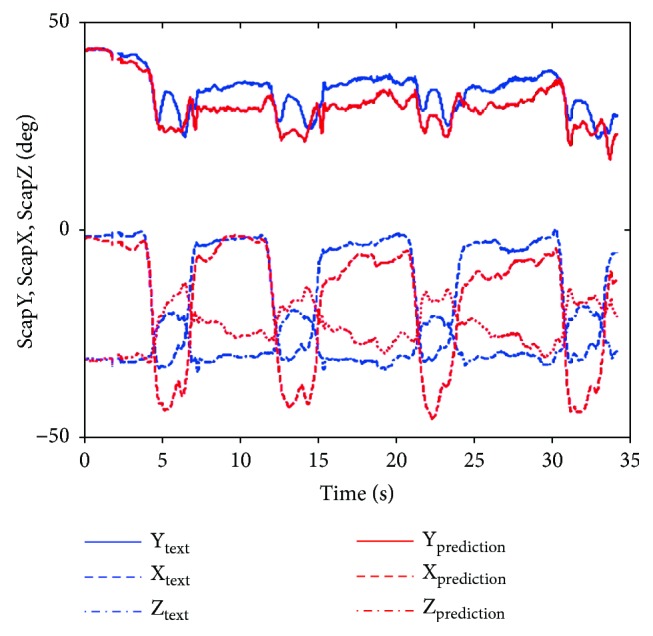
The movement of the scapula with respect to the thorax (ABD).

**Figure 15:**
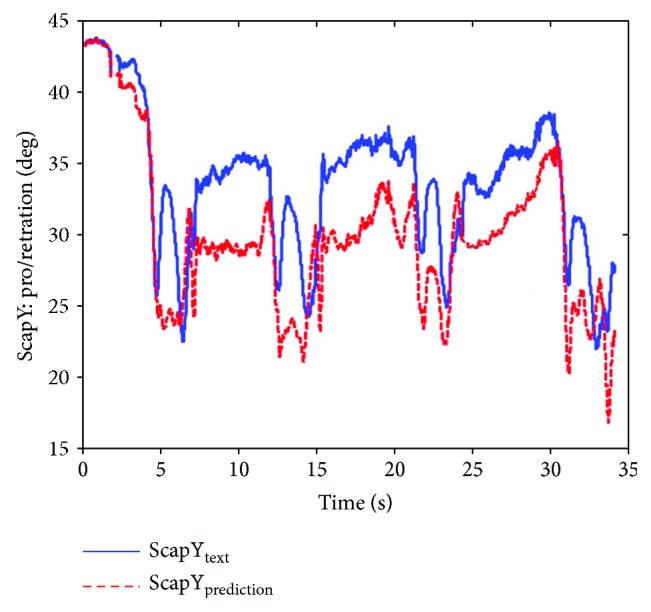
Comparison of protraction obtained by prediction and measurement (ABD).

**Figure 16:**
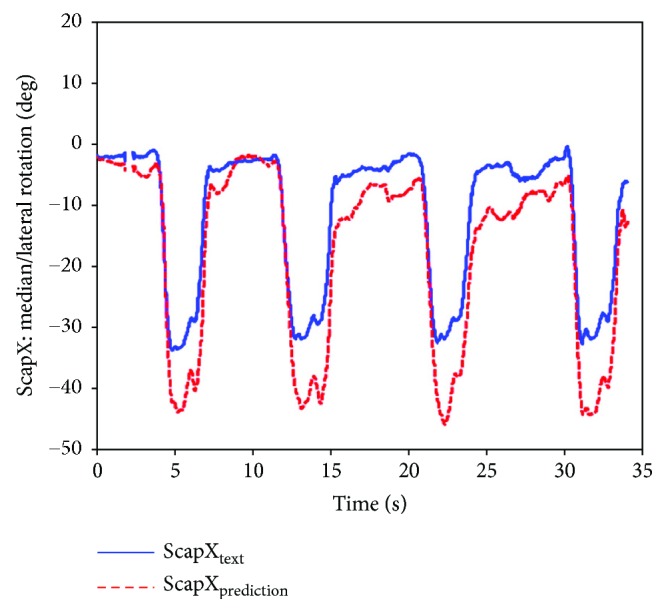
Comparison of lateral rotation obtained by prediction and measurement (ABD).

**Figure 17:**
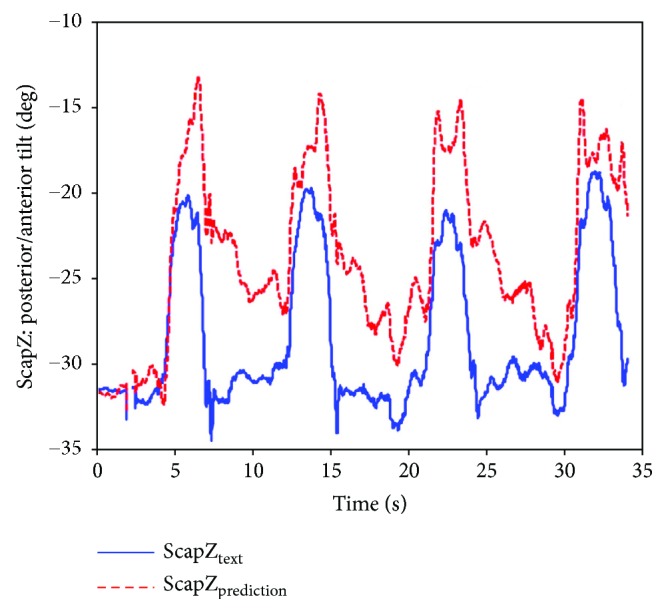
Comparison of anterior tilt obtained by prediction and measurement.

Thus, the shoulder girdle mechanism position analysis is finished, and the results in the calculated coordinate system of the bone or joint movement posture can be obtained and then converted corresponding to the ISB standard system, so the agency model for shoulder skeletal motion prediction results can be obtained and compared with the experimental measurement results.  Figure 14 shows the scapula relative to the thorax motion posture analysis results (ST joint). Figures 15, 16, and 17 are the results of the components of the scapula posture matrix after the decomposition of YXZ rotation sequence according to the ISB standard. Thanks to the fruitful achievement in biological research, the detail and accurate experimental data on shoulder motion can be provided, and in this paper, we selected a set of representative data to verify that the kinematic description of the skeleton model and experimental measurements are consistent.

Using the same method, the exercise experiments of FLEX and SCAP can be analyzed, and the agency model shoulder motion configuration prediction and experimental measurement results are basically consistent.

## 3. Shoulder Rhythm Model

From the view of the mechanistic model, the three rotational degrees of freedom of the humerus are independent from the three degrees of freedom of movement of the shoulder girdle mechanism. However, a great number of studies have shown that when the shoulders do some basic movements in the natural state (such as abduction, adduction, external rotation, pronation, flexion, and anteversion), there is a significant correlation and repeatability of shoulder girdle and humeral motion, following the law of the rhythm of the shoulder joint.

This article simplifies shoulder joint rhythm analysis to the problem of establishing the relationship between the humeral posture and the three independent input joint variables of the shoulder girdle mechanism.

### 3.1. Rhythm Analysis of Three Kinds of Exercise Task Experiment

The shoulder rhythm of an individual is highly repeatable and less affected by the size of the load when shoulders are in the condition of lighter load [27, 28], but there exist individual differences [29]. Therefore, this paper extends the traditional shoulder joint rhythm and describes the relationship between the scapula and the clavicle posture and the posture of the humerus individually via the proposed model with three independent joint variables (*θ*_1_, *θ*_2_, *ϕ*_*d*_). Through the constraints in the proposed model considering the individual size of shoulder complex, three independent joint variables can be further used to determine the corresponding relationship between the entire shoulder girdle posture and the humeral posture. This method reduces the number of variables involved in rhythms and increases the impact of individual differences.

Studies of shoulder rhythms by the literature [17, 25–28] showed that the elevation plane (HumY_1_) and the elevation (HumX) are relatively independent in shoulder bone movement, while the shoulder girdle posture with two joint variables has a strong regularity. When modeling the shoulder rhythms, this research also uses planes of elevation and elevation as independent variables for rhythm functions.

#### 3.1.1. ABD Sports Rhythm

**Figure 18:**
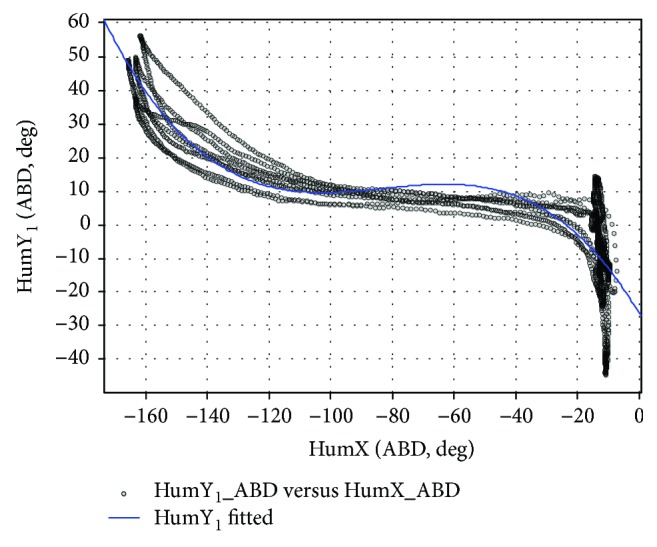
The relation between plane of elevation angle and elevation angle (ABD).

During the ABD exercise, five actions of lifting and dropping humerus are finished, as shown in Figure 6. Although the subject was asked to do abduction in the frontal plane, the actual elevation of the plane mainly focuses in the scoped of −10° to 20°, as shown in Figure 18. Although when the shoulder moved, the joint variable of elevation plane and the joint variable of elevation are considered independent from each other, the strong regularity and reflexivity are shown in Figure 18, because it represents the physical coordination of the experimental subjects in a certain motion like ABD exercise, and the coupling phenomenon will disappear in arbitrary motion. The relationship between the elevation plane and the elevation in Figure 8 is not universal, so this article will use the plane of elevation and the elevation as two independent variables of the law function.

**Figure 19:**
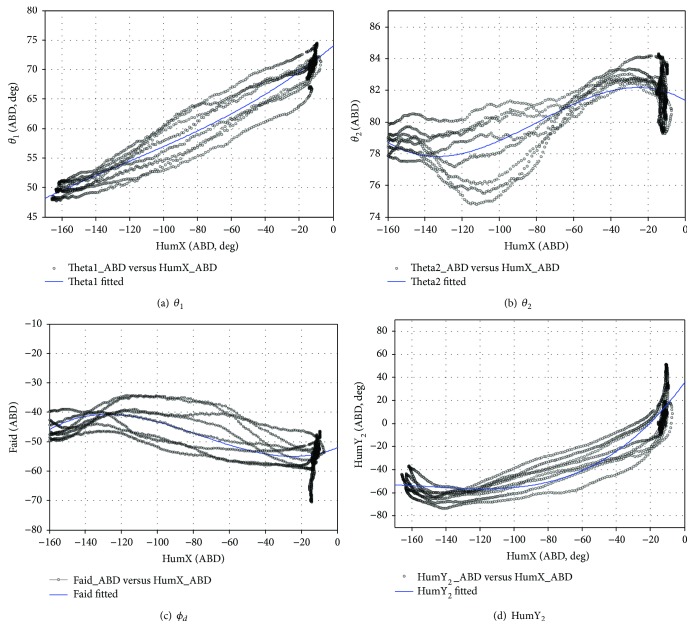
The joint angles are fit using a third-order polynomial (ABD).

Figure 19 shows the variation of various dependent variables with the humerus elevation angle when the subject does ABD exercise. *θ*_1_, *θ*_2_, *ϕ*_*d*_ is used to determine the rhythm of the shoulder girdle, while Figure 19(d) is used to determine the rhythm of humerus rotation around its shaft (HumY_2_, internal/external rotation). Cubic polynomial fitting is adopted, and shoulder rhythm function can be obtained:

(26) $$\begin{array}{c} \left[\begin{array}{c} \theta_{1}\\ \theta_{2}\\ \phi_{d}\\ {HumY}_{2} \end{array}\right]=\left[\begin{array}{c} {\left[P_{\theta_{1}}\right]}_{1\times 4}\\ {\left[P_{\theta_{2}}\right]}_{1\times 4}\\ {\left[P_{\phi_{d}}\right]}_{1\times 4}\\ {\left[P_{{HumY}_{2}}\right]}_{1\times 4} \end{array}\right]\left[\begin{array}{c} {\left(HumX\right)}^{3}\\ {\left(HumX\right)}^{2}\\ \left(HumX\right)\\ 1 \end{array}\right], \end{array}$$

where ${\left[P\right]}_{1\times 4}=\left[\begin{array}{cccc} p_{1} & p_{2} & p_{3} & p_{4} \end{array}\right]$ denotes the polynomial fitting coefficients to the corresponding joint variables. For simplifying the calculation, the rhythm function corresponding to the ABD motion is expressed as follows:

(27) $$\begin{array}{c} f_{ABD}\left(HumX\right)=p_{1}{\left(HumX\right)}^{3}+p_{2}{\left(HumX\right)}^{2}+p_{3}\left(HumX\right)+1. \end{array}$$

Table 5 shows the fitting results of the dependent variables, and the accuracy and goodness of the fitting curve are, respectively, measured by root mean square error (RMSE) and coefficient of determination (*R*^2^).

#### 3.1.2. SCAP Sports Rhythm

During the entire SCAP exercise, lifting and dropping actions in the humerus were completed 4 times. To analyze the ABD motor rhythm, a rhythm function is constructed.

(28) $$\begin{array}{c} f_{SCAP}\left(HumX\right)=p_{1}{\left(HumX\right)}^{3}+p_{2}{\left(HumX\right)}^{2}+p_{3}\left(HumX\right)+1. \end{array}$$

**Figure 20:**
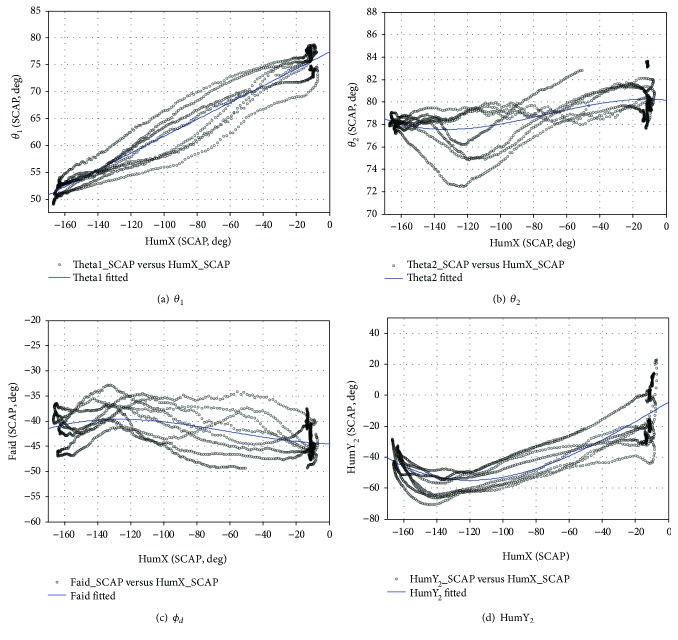
The joint angles are fit using a third-order polynomial (SCAP).

Figure 20 shows the fitting result curve, and Table 6 shows the polynomial rhythm function coefficients, RSME, and coefficients of determination.

#### 3.1.3. FLEX Sports Rhythm

During the entire FLEX exercise, lifting and dropping actions in the humerus were completed 4 times. The same as the ABD motor rhythm analysis, a rhythm function is constructed.

(29) $$\begin{array}{c} f_{FLEX}\left(HumX\right)=p_{1}{\left(HumX\right)}^{3}+p_{2}{\left(HumX\right)}^{2}+p_{3}\left(HumX\right)+1. \end{array}$$

**Figure 21:**
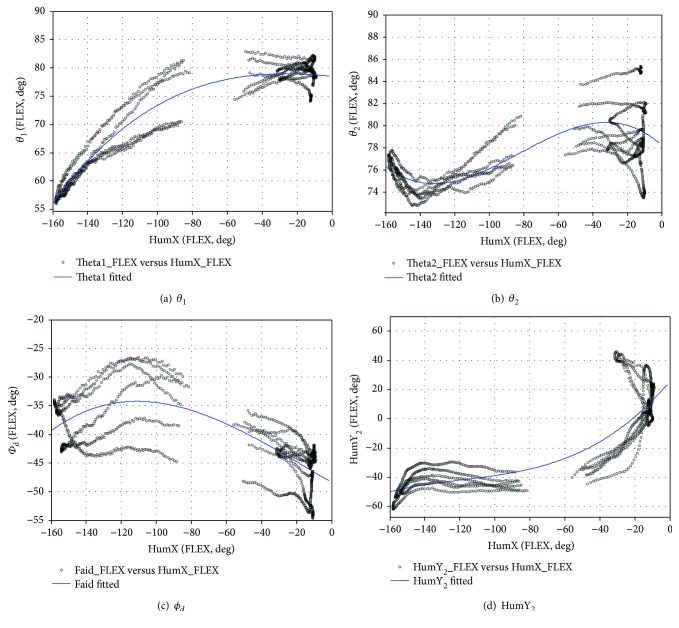
The joint angles are fit using a third-order polynomial (FLEX).

Figure 21 shows the fitting result curve, and Table 7 shows the polynomial rhythm function coefficient, RSME, and coefficient of determination.

### 3.2. Shoulder Rhythm Expansion

**Figure 22:**
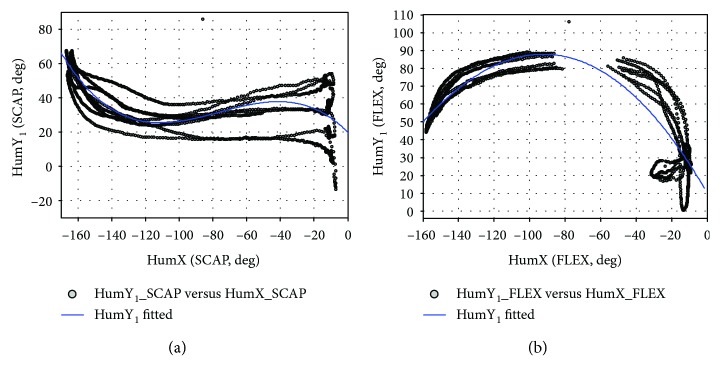
The ranges of plane of elevation when the movement of SCAP and FLEX is exerted.

The lifting plane is not uniquely fixed during ABD, SCAP, and FLEX movements. In the ABD, SCAP, and FLEX, the humerus lifting plane angle HumY_1_ changes as shown in Figures 16 and 22. It can be seen that the main movement range of the plane of elevation during these three movements is as shown in Table 8.

Theoretically, the variables of the shoulder joint rhythm function are HumY_1_ and HumX in the whole range of activity, and the relationship between the movement of the shoulder bones and the axial rotation angle HumY_2_ of the humerus is not significant. At the same time, axial rotation angles HumY_2_ and HumX are related. Therefore, in this research, the shoulder joint rhythms corresponding to ABD, SCAP, and FLEX are extended to the motion space −10° ≤ HumY_1_ ≤ 90° and 0° ≤ HumX ≤ 165°, and the rhythm can be expressed as follows:

(30) $$\begin{array}{c} f\left(HumX\right)=\left\{\begin{array}{c} f_{ABD}\left(HumX\right),\;-10^{{}^{\circ}}\leq {HumY}_{1}\leq 20^{{}^{\circ}},\\ f_{SCAP}\left(HumX\right),\;20^{{}^{\circ}}\leq {HumY}_{1}\leq 50^{{}^{\circ}},0^{{}^{\circ}}\\ f_{FLEX}\left(HumX\right),\;50^{{}^{\circ}}\leq {HumY}_{1}\leq 90^{{}^{\circ}}. \end{array}\right.\leq HumX\leq 165^{\circ }, \end{array}$$

## 4. Conclusion

In this paper, a new shoulder complex skeletal model based on joint geometric constraints is proposed using a spatial hybrid mechanism to describe the movement of human shoulder complex, which is developed for the rehabilitation robots. The effectiveness and viability of the proposed model have been verified via the experimental data of shoulder skeletal motion. Based on this model, through the analysis of the angle and change rule of each joint in the shoulder complex during the movement, the shoulder rhythm was obtained by means of polynomial fitting with HumY_1_ and HumX as independent variables to make it suitable to guide the training strategy for shoulder rehabilitation via robotics.

## Figures and Tables

**Table 1:** The size parameters of each link of mechanism model.

*l*_1_	*l*_2_	*l*_3_	*l*_22_	*l*_4_	*γ*
154.17 mm	120.71 mm	187.21 mm	109.83 mm	301.56 mm	33.8°

**Table 2:** The size parameters of thorax ellipsoid (mm).

*m*	*n*	*p*	^0^ *p*_o_
76.85	97.17	217.44	${\left[\begin{array}{cccc} 28.12 & -43.90 & -164.51 & 1 \end{array}\right]}^{T}$

**Table 3:** The skeletal feature size parameters of the experimental subjects (mm).

Bone markers	Common parameters	Test subject
IJ	(0, 0, 0)	(0, 0, 0)
PX	(31.9, −132.7, −9.8)	(56.0, −163.0, −2.4)
C7	(−124.2, 54.1, 0.0)	(−123.1, 80.7, −4.7)
T8	(−156.61, −171.5, 0.0)	(−179.1, −133.2, 11.2)
AA	(−105.6, 7.5, 182.6)	(−73.5, 9.3, 205.1)
TS	(−156.0, −11.7, 75.0)	(−136.3, 5.1, 121.8)
AI	(−156.7, −126.2, 101.9)	(−141.6, −126.5, 116.2)
AC	(−71.8, 26.6, 165.1)	(−37.0, 40.4, 167.2)
SC	(−2.8, −15.2, 1.4)	(15.3, −31.0, −6.6)
Ellipsoid center	(−62.1, −152.1, 0.0)	(−61.6, −148.1, 0.0)
Ellipsoidal semimajor axis	(95.6, 211.7, 144.6)	(114.4, 222.7, 164.8)
Zoom factor		(1.20, 1.05, 1.14)

**Table 4:** Range of motion (ROM) tasks.

Shoulder movement	Motion description
ABD (shoulder abduction)	Motion in the frontal plane. Initially, the arm relaxes and hangs down and then moves in the frontal plane, until the humerus rises up to the extreme position, and then moves back to the initial location. Keep the palm facing the body and upper limb unbent during exercises.
FLEX (shoulder anteflexion)	Flexion movement in the sagittal plane. Initially, the arm relaxes and hangs down and then flexes in the sagittal plane, until the humerus rises up to the extreme position, and then moves back to the initial location.
SCAP (shoulder scaption)	Movement in the scapular plane. Initially, the arm relaxes and hangs down and then raises the scapular plane (at about 45° to the frontal plane) until the humerus rises up to the extreme position and then back to the initial position.

**Table 5:** Third-order polynomial fit parameters of the rhythm function (ABD).

Dependent variable joint angle	Polynomial coefficients				*R*-square	Deg
	*p*_1_	*p*_2_	*p*_3_	*p*_4_	*R* ^2^	RMSE
*θ*_1_	2.483*e* − 06	0.0009501	0.2419	74.07	0.9581	1.811
*θ*_2_	−6.441*e* − 06	−0.001558	−0.06742	81.4	0.6004	1.271
*ϕ*_*d*_	2.822*e* − 05	0.006688	0.3163	−50.89	0.5203	4.848
HumY_2_	2.443*e* − 05	0.01201	1.833	32.07	0.8376	13.76

**Table 6:** Third-order polynomial fit parameters of the rhythm function (SCAP).

Dependent variable joint angle	Polynomial coefficients				*R*-square	Deg
	*p*_1_	*p*_2_	*p*_3_	*p*_4_	*R* ^2^	RMSE
*θ*_1_	−6.283*e* − 06	−0.001732	0.03207	76.14	0.9574	1.922
*θ*_2_	−2.328*e* − 07	−3.272*e* − 06	0.01831	80.13	0.9161	0.6043
*ϕ*_*d*_	4.478*e* − 06	0.0008388	−0.002783	−44.38	0.3585	2.969
HumY_2_	−1.417*e* − 05	8.118*e* − 05	0.6336	4.591	0.6418	12.61

**Table 7:** Third-order polynomial fit parameters of the rhythm function (FLEX).

Dependent variable joint angle	Polynomial coefficients				*R*-square	Deg
	*p*_1_	*p*_2_	*p*_3_	*p*_4_	*R* ^2^	RMSE
*θ*_1_	3.587*e* − 06	−0.000406	−0.01044	79.29	0.9963	0.475
*θ*_2_	−1.058*e* − 05	−0.002583	−0.1365	77.43	0.3921	2.833
*ϕ*_*d*_	6.167*e* − 06	0.0002202	−0.179	−48.39	0.5784	3.951
HumY_2_	3.011*e* − 05	0.01069	1.412	25.64	0.7932	12.75

**Table 8:** The ranges of plane of elevation.

Type of exercise	ABD	SCAP	FLEX
Range of plane of elevation	−10°~20°	20°~50°	50°~90°
